# Segmented-overlap Fourier filtering and averaging (SOFFA) approach to improve concentration sensitivity of magnetic resonance spectra

**DOI:** 10.5194/mr-7-99-2026

**Published:** 2026-07-02

**Authors:** Jason W. Sidabras

**Affiliations:** 1 Medical College of Wisconsin, Department of Biophysics, 8701 Watertown Plank Rd, Wauwatosa, WI 53226, USA

## Abstract

The segmented-overlap Fourier filtering and averaging (SOFFA) data acquisition method is described in detail for magnetic resonance spectroscopy. In this work, the four processes that encompass the SOFFA data acquisition method are detailed: (i) oversampling spectral segments, (ii) Fourier block-filtering, (iii) segment-overlap averaging, and (iv) decimation. Three experimental examples are shown: first, conventional continuous-wave (CW) electron paramagnetic resonance (EPR) is compared to SOFFA-CW of a single reduced $[4\mathrm{Fe}\text{-}4S{]}^{+}$ ( S=1/2 ) at concentrations of 1 mM, 100 µM , and 10 µM , showing an average increase in concentration sensitivity by a factor of 5.6 in a 100 min measurement time. Second, an experimental comparison of CW and SOFFA non-adiabatic rapid-scan (SOFFA-NARS) data with similar filter parameters and field modulation amplitude demonstrates a factor of 10.3 in signal-to-noise (SNR) improvement (32 min measurement time) for a 150 µM site-directed spin-labeled hemoglobin in 82 % glycerol at 18 °C . Finally, an SNR-matched experiment of free TEMPO at 10 µM concentration is presented, where CW was performed at 400 scans (273 min) compared to SOFFA-CW with an overlap factor of 100 (20 min). Also shown is the effect of 1/f noise on these CW and SOFFA-CW experiments. Ultimately, the SOFFA algorithm achieves sensitivity enhancement by combining massive digital oversampling and out-of-band noise filtration with coherent spatial accumulation of highly overlapped spectral segments. The gains reported here are phenomenological and are grounded in established digital signal processing principles but are validated through experiments rather than closed-form analytical prediction. This fundamental restructuring of the acquisition chain successfully decouples high-frequency filtering from low-frequency averaging while providing a data collection scheme that suppresses 1/f noise. The SOFFA method can be implemented to perform real-time segmented processing and, combined with more sophisticated averaging methods, will push the state-of-the-art sensitivity in magnetic resonance spectroscopy.

## 1 Introduction

**Figure 1:**
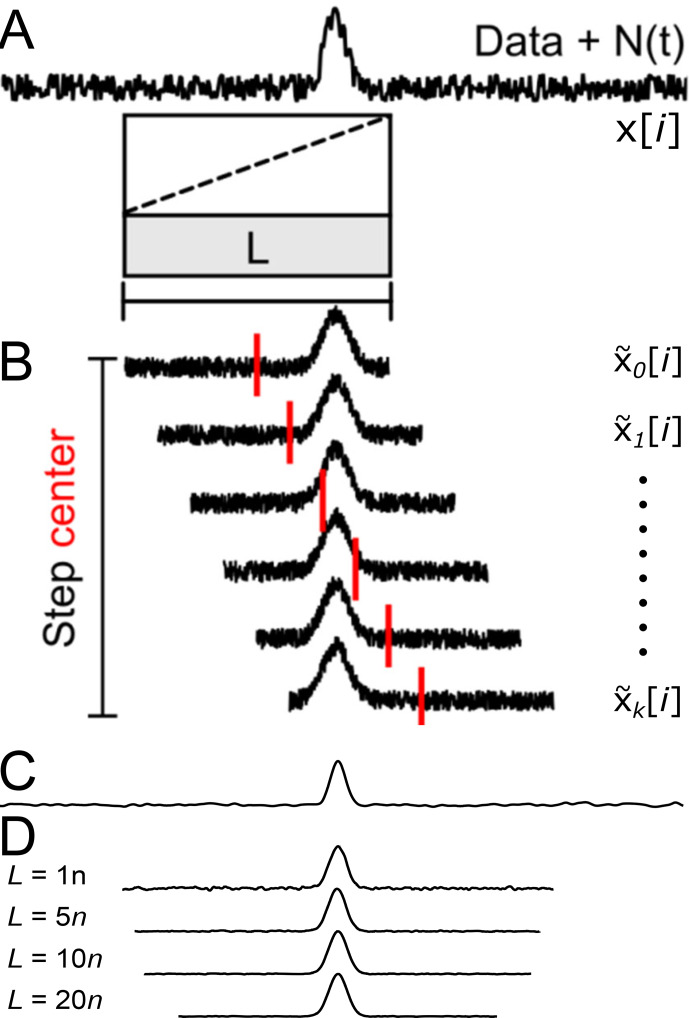
A visualization of the SOFFA method. A Gaussian absorption spectrum is simulated with a half width of 1 mT and an amplitude of unity. Shown here is a representation of the simulated experiment. **(A)** Random (white) noise is added. **(B)** A small set of unfiltered segmented data is plotted, showing field steps s with oversampling. **(C)** When a Gaussian convolution filter of Eq. (6) is applied to **(A)**, the expected improvement in SNR is shown, similarly to increasing the time constant. **(D)** However, using the SOFFA method, L is increased as a function of n , where n is in points. The discrete shift between adjacent segments, of sweep size s in mT, leads to further SNR improvement being exhibited due to the increased averaging during concatenation of filtered data.

In this work, the segmented-overlap Fourier filtering and averaging (SOFFA) method for magnetic resonance data acquisition and filtering is established. In a typical continuous-wave (CW) electron paramagnetic resonance (EPR) experiment, a spectrum is collected as a continuous signal; i.e., the spectroscopist must “play” it until the end and, only then, repeat for averaging. However, the signal is typically time invariant and therefore can be collected in a number of unique ways. It is shown herein that, by collecting a magnetic resonance spectrum in segments, an additional parameter related to the overlap of each scan is introduced while increasing the available number of data points collected beyond hardware limitations. Each acquired segment contains a field- or frequency-stepped correlated signal and uncorrelated noise. For processing, each segment is oversampled, filtered block-wise, concatenated, and decimated to a standard number of points (i.e., 1024), resulting in a signal-to-noise (SNR) improvement compared to traditional CW or non-adiabatic rapid-scan (NARS) data collection, averaging, and filtering methods for the same measurement time. The SOFFA data acquisition method can be adopted for experiments with a swept parameter, correlated signal, and uncorrelated noise. Here, this is demonstrated for conventional CW and NARS techniques. A visualization of the scheme is illustrated in Fig. 1.

Illustrated in Fig. 1A, is a representative typical CW-EPR spectrum of a Gaussian signal with white noise. After standard filtering, the SNR ratio is improved, as shown in Fig. 1C. For SOFFA, the spectrometer acquires a spectrum by stepping a narrow sweep window L across the full field range in overlapping segments and then maps each segment onto a common high-resolution grid via overlap-add accumulation, shown in Fig. 1B. Each segment is individually filtered in the frequency domain before being re-accumulated and averaged across the overlapping regions. The resulting high-resolution grid is then smoothed and decimated to the desired output point count, yielding a final spectrum with substantially improved SNR compared to a single full-width sweep. Improvement of the SNR depends on the step size n , which increases the averages due to overlapping m . The overlap between segments is the key feature: it provides redundant sampling at every field position, and so the averaging step coherently reinforces the signal while averaging down incoherent noise.

The improved SNR ratio achieved by the SOFFA method can be leveraged to reduce the time required for an experiment, thereby increasing throughput. In traditional EPR experiments, SNR improves as $\sqrt{m_{a}}$ with the number of averages $m_{a}$ , meaning that doubling the SNR requires quadrupling the experiment time. With the SOFFA method, the same SNR level can, in principle, be achieved in a fraction of that time by exploiting the overlap averaging m and segment filtering rather than brute-force averaging. However, a practical minimum experiment time exists for SOFFA-CW that is not present in conventional averaging.

The limitations of SOFFA are that the per-segment sweep rate must remain slow enough to avoid passage (Weger, 1960), and the magnet step size is bounded by the field controller precision and hysteresis of the electromagnet, with practical step sizes on commercial instruments being limited to 0.01 mT . Together, these constraints set a floor on the total acquisition time and a ceiling on the number of averages due to overlapping m , which cannot be increased without violating one of the hardware limits. Within those bounds, SOFFA decouples the SNR-per-unit-time relationship of conventional averaging. These two practical hardware limitations (minimum step size and maximum sweep rate) are largely eliminated using the non-adiabatic rapid scan (NARS) method (Kittell et al., 2011; Kittell et al., 2012; Kittell and Hyde, 2015; Hyde et al., 2013; Yu et al., 2015).

In a NARS experiment, the field or frequency is swept at a rate and amplitude that allows the spin system to return to thermal equilibrium, and the pure-absorption lineshape is recorded. In this sense, NARS mimics a CW EPR experiment, and the power saturation profile of the sample remains unperturbed. Typically, NARS experiments employ a low amplitude (1 mT ) and rate (below 50 kHz) for field or frequency sweeps. The increased rate and amplitude at a fixed field position of a NARS experiment generate harmonics which are captured completely with a digitizer. In practice, the spectrum is collected in segments by stepping the static field to obtain the full spectrum. Each step is windowed, zero-filled, concatenated, and filtered. The advantage of NARS comes from the modern data collection methods (averaging, digital filtering, etc.), collection of pure-absorption spectra, and SNR improvements due to overlapping segments during concatenation. The NARS method collects quadrature pure-absorption EPR spectra, which can be pseudo-modulated to the conventional first-derivative EPR spectrum (Hyde et al., 1990; Hyde et al., 2013). NARS has been used by Hyde and colleagues (Kittell et al., 2011; Kittell and Hyde, 2015) at L-band (1.2 GHz) to extend the deconvolution method of CW EPR distance determination from 18–25 to 30–35 Å (Kittell et al., 2012) and has been used to increase the resolution for a copper imidazole complex (Hyde et al., 2013). Additionally, NARS was independently shown by Eaton and colleagues in applications of spectra as wide as 620 mT using concatenation without overlap (Yu et al., 2015).

It has been established that NARS data collection produces a comb filter that suppresses high-frequency noise (Wilmshurst, 1990; Eaton et al., 2014). However, these methods are susceptible to low-frequency noise that is correlated with the field sweep or the transfer function of the frequency response of the system. Filtering such noise is problematic since the features may contain the same frequency content as the absorption spectrum. Pseudo-modulation using the moving-difference (MDIFF) algorithm to calculate the conventional first-harmonic derivative-like spectrum further reduces some low-frequency noise (Hyde et al., 2013). However, the challenge of properly filtering NARS data still remains. The SOFFA data acquisition method is shown to remove challenging noise profiles, and the method is well suited for magnetic resonance spectroscopy with minimum changes to data acquisition.

It should be noted that the SNR improvements reported herein are phenomenological in character. While the SOFFA method is constructed from well-established DSP principles (oversampling theory, Fourier block-filtering, overlap-add averaging, and decimation), a complete closed-form analytical expression that quantitatively predicts the net gain from the combination of all four processing stages is beyond the scope of this work. The experimental results serve as the primary validation. Future work will focus on a rigorous theoretical framework that decomposes the contributing factors and yields quantitative predictions as a function of acquisition parameters.

In this work, the SOFFA data acquisition method is tested with (i) an experimental comparison between an averaged and filtered CW spectrum and a SOFFA-CW spectrum using concentrations of 1 mM, 100 µM , and 10 µM of apo [FeFe]-hydrogenase (single reduced $[4\mathrm{Fe}\text{-}4S{]}^{+}$ ; S=1/2 ; (Knörzer et al., 2012)); (ii) an experimental comparison of a SOFFA-NARS spectrum of 150 µM site-directed spin-labeled hemoglobin protein in 82 % glycerol at 18 °C to averaged and filtered CW spectrum; and (iii) an experimental comparison between CW and SOFFA-CW using a 10 µM TEMPO (2,2,6,6-tetramethylpiperidine-1-oxyl) radical in water where SNR is matched.

## 2 Methods

### 2.1 Continuous-wave EPR

Three concentrations of apo [FeFe]-hydrogenase from *Chlamydomonas reinhardtii* (single reduced $[4\mathrm{Fe}\text{-}4S{]}^{+}$ , S=1/2 ) were prepared at 1 mM, 100 µM , and 10 µM and confirmed by UV-VIS absorption at 400 nm (Knörzer et al., 2012). All spectra were collected at 15 K with an incident microwave power of 0.63 mW and a field modulation amplitude of 0.5 mT at 100 kHz. The 1 mM reference spectrum was collected as a 4 min scan over 100 mT with 4096 points averaged 9 times. The 100 µM and 10 µM concentrations were each collected with 25 averages (4 min scan, 100 mT , 4096 points) for a total measurement time of 100 min.

For the SOFFA-CW experiments, the center field was stepped in 0.5 mT increments over a total sweep of 100 mT , yielding K=200 steps, with a segment sweep width g=25mT and 4096 points per segment. Each segment was collected over 30 s for a total measurement time of 100 min. Segments were acquired using a ProDEL script in Bruker Xepr (v.2.6b-160), which steps the center field and stores each segment in Bruker DSC/DTA format.The ProDEL script and Python GUI used to collect and process SOFFA data are available at https://github.com/jsidabras/SOFFABruker (last access: 18 June 2026). The ProDEL script iterates discrete static field steps, triggers acquisition at each position, and saves the resulting 2D dataset. Software overhead is negligible; the acquisition rate is limited by the hardware field-settling time of the magnet between steps. This approach has been validated for field sweeps up to 8192 points with 1200 steps (0.01 mT step size over 12 mT ).

The single reduced $[4\mathrm{Fe}\text{-}4S{]}^{+}$ CW EPR spectra and complementary SOFFA-CW EPR spectra were collected on a Bruker E580 Elexsys X-band spectrometer (9.70 GHz) equipped with a bismuth germanate ( ${\mathrm{Bi}}_{4}({\mathrm{GeO}}_{4}{)}_{3}$ , BGO) dielectric resonator retrofitted in a Bruker MD5 housing (Ivanov et al., 2016).

The 10 µM TEMPO spectra were recorded on a Bruker E500 Elexsys II X-band spectrometer equipped with a Super High-Q cylindrical ${\text{TE}}_{011}$ model (no. E4122011). The traditional CW EPR was measured with a 10 mT sweep of 1024 points over 41.94 s with 400 averages, 20.48 ms time constant at 3.2 mW , and 0.1 mT field modulation at 100 kHz. SOFFA-CW was performed with a 3 mT sweep of 8192 points over 10.39 s, with 100 steps of 0.1 mT each, a 1.28 ms time constant at 3.2 mW , and 0.1 mT field modulation at 100 kHz.

### 2.2 Non-adiabatic rapid scan

The conventional CW comparison and NARS spectra were collected on a custom-built X-band bridge dedicated to segmented-NARS spectroscopy (Hyde et al., 2022). A 150 µM site-directed spin-labeled hemoglobin sample in 82 % glycerol was measured at 18 °C in a five-loop–four-gap loop–gap resonator at 9.81 GHz. For the conventional CW reference, 16 averages of 2 min scans were collected over 20 mT with 0.1 mT field modulation at 100 kHz, a 5 ms time constant, and 1 mW of incident microwave power. The 5 ms time constant was chosen to minimize lock-in pre-filtering within each scan, preserving the full signal bandwidth for subsequent filtering post-processing. For the SOFFA-NARS experiment, the static field was stepped in 0.025 mT increments with an overlap of m=40 using a trapezoidal field ramp at 11.33 kHz with a 50 µs flat region and an amplitude of 0.5 mT . Data were acquired over 95 % of the trapezoidal sweep in one direction; the resulting edge effects appear as periodic noise outside the signal power spectral density and are suppressed by segment-overlap averaging. Both experiments required approximately 32 min of measurement time.

### 2.3 SNR definition

The SNR is defined as

1 $$\text{SNR}=\frac{S_{\text{pk}}}{\sigma_{\text{noise}}},$$

where $S_{\text{pk}}$ is the peak-to-peak signal amplitude, and $\sigma_{\text{noise}}$ is the standard deviation of the noise, computed from an off-resonance region of the spectrum (Schroeder, 2000; Oppenheim et al., 1999).

## 3 Theory and implementation

**Figure 2:**
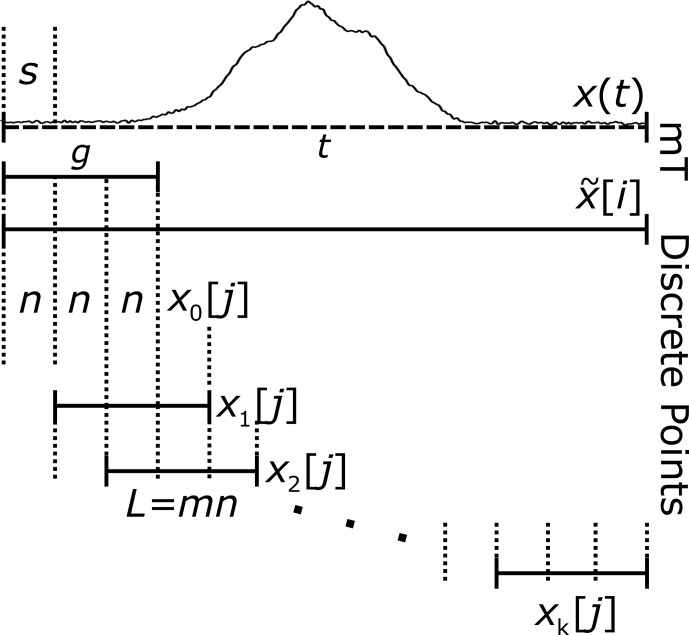
Illustration of spectral segmentation with parameters and variables.

The SOFFA data acquisition method consists of four distinct processing stages: (i) oversampling spectral segments, (ii) Fourier block-filtering, (iii) segment-overlap averaging, and (iv) decimation. The nomenclature of the Welch fast Fourier transform (FFT) procedure for power spectral density estimation (Welch, 1967) is adopted throughout due to the similarity of spectral segmentation to that procedure. Table 1 defines all symbols used in this work, with a graphic representation in Fig. 2.

**Table 1:** Principal variables and symbols used in this work.

Symbol	Definition	Units
Signal representation		
x(t)	Continuous noisy spectrum in the swept domain	–
$\tilde{x}[i]$	Discretized full-spectrum signal on the conventional grid	–
S(t)	True signal component	–
N(t)	Additive noise component (white or 1/f )	–
Acquisition and segmentation		
t	Total spectral sweep width	mT
g	Segment sweep width	mT
s	Field step size between adjacent segment starts	mT
K	Total number of acquired segments (or segment positions)	–
N	Number of points in a conventional full-spectrum acquisition	pts
L	Number of points retained in each segment window	pts
n	Discrete shift between adjacent segment starts on the fine grid	pts
k	Segment index, k=0,1,…,K-1	–
$x_{k}[j]$	k th acquired segment, j=0,1,…,L-1	–
m	Overlap factor between neighboring segments	–
$t_{e}$	Total experiment time	s
τ	Acquisition time per collected trace	s
$m_{a}$	Number of conventional averages	–
Fourier filtering and accumulation		
ω	Discrete frequency index in Fourier space	–
γ	Center point of the Gaussian filter in Fourier space	–
$\sigma_{n}^{2}$	Gaussian filter variance	–
${\text{H}}_{k}[\omega ]$	Gaussian filter applied to segment k	–
${\text{Y}}_{k}[\omega ]$	Fourier transform of the zero-filled, field-aligned k th segment	–
${\text{G}}_{k}[\omega ]$	Filtered Fourier-domain segment	–
$y_{\text{tot}}[i]$	High-resolution concatenated spectrum before final decimation	–
Final output and SNR quantities		
P	Number of points in the final decimated SOFFA spectrum	pts
y[p]	Final decimated SOFFA spectrum, p=0,1,…,P-1	–
$S_{\text{pk}}$	Peak-to-peak signal amplitude	–
$\sigma_{\text{noise}}$	Standard deviation of the off-resonance noise	–
OSR	Oversampling ratio	–
$f_{s}$	Effective sampling frequency	Hz
$f_{\text{cw}}$	Spectral bandwidth of the conventional CW experiment	Hz
$\tau_{c}$	Effective time constant of the oversampled acquisition	s
$\tau_{\text{cw}}$	Conventional CW time constant	s

Here, linear noise is assumed, such that,

2 $$\tilde{x}(t)=S(t)+N(t),$$

where S(t) is the signal that is detected, and N(t) is either spectrally flat (white) noise, characterized by a frequency-independent power spectral density, or 1/f (pink) noise, characterized by a power spectral density that increases with decreasing frequency. Noise that is a function of the signal is beyond the scope of this work.

For conventionally acquired data, the number of points, scan rate, and time constant are chosen to represent the spectrum completely and without spectral distortions; simulated data for visualization are shown in Fig. 1A. For each new scan, the noise is uncorrelated; therefore, the data can be averaged and filtered. For example, filtered data of Fig. 1A are shown in Fig. 1C. Fourier filtering averaged data before or after summing the individual scans are equivalent. With traditional averaging and white noise, the SNR follows a strict $\sqrt{m_{a}}$ gain, where $m_{a}$ is the number of averages. The same signal–time trade-off exists when increasing the number of collected data points while maintaining equivalent measurement time.

In the following sections, the four processes utilized to create the SOFFA data acquisition method are detailed: (i) oversampling spectral segments, (ii) Fourier block-filtering each segment separately, (iii) segment-overlap averaging, and (iv) decimation. It is the combination of these processes that improves the SNR ratio for the same amount of data collection time.

### 3.1 Oversampling spectral segments

In the SOFFA data acquisition method, each field- and/or frequency-swept segment has the following properties: (i) the overlapping noise is non-coherent, (ii) field- or frequency-dependent features are considered to be the signal, and (iii) each segment is an oversampled section of the whole spectrum. Here, oversampled means that each SOFFA segment is acquired with substantially more points than what is required to represent the underlying spectral features at the target final resolution.

In the case of the SOFFA method, all segments are collected separately and can then be further windowed to the desired length to remove potential edge effects with the window size L . Stepping the field by s over the spectrum yields k individually collected segments. The step size s is represented by the discrete shift step size n , illustrated in Fig. 2. Since magnetic resonance produces a stationary and time-invariant signal, the corresponding output blocks can be zero-filled by a length of the total collection size to begin at the same absolute step size, aligning the field- or frequency-dependent features. The zero-fill parameter is then shift-stepped by n to align field-dependent features. To maximize the overlap averaging after filtering, the windowing function is chosen to be rectangular, and no aliasing issues exist if oversampling is used. Unlike windowing in typical block-filtering, which benefits greatly from window choice (Harris, 1978), a rectangular window will maximize the individual contribution of each segment during the concatenation process. Any higher-order frequencies generated by discontinuities are removed in the filtering process and result in proper concatenation and aliasing.

In the SOFFA data acquisition method, each segment is oversampled in order to reduce the noise spectral density while keeping the signal spectral density constant (Oppenheim et al., 1999). One can estimate an effective time constant,

3 $$\tau_{c}=\frac{1}{f_{s}}=\frac{t_{e}}{nK},$$

where $f_{s}$ is the effective sampling frequency, $t_{e}$ is the total experiment scan time, n is the shift step size, and K is the total number of segments. If the noise is predominantly white and a perfect low-pass filter is used, the SNR is improved by the oversampling ratio,

4 $$\text{OSR}=\frac{f_{s}}{2f_{\text{cw}}},$$

where $f_{\text{cw}}$ is the inverse of the time constant $\tau_{\text{cw}}$ of the conventional CW data collection.

**Figure A1:**
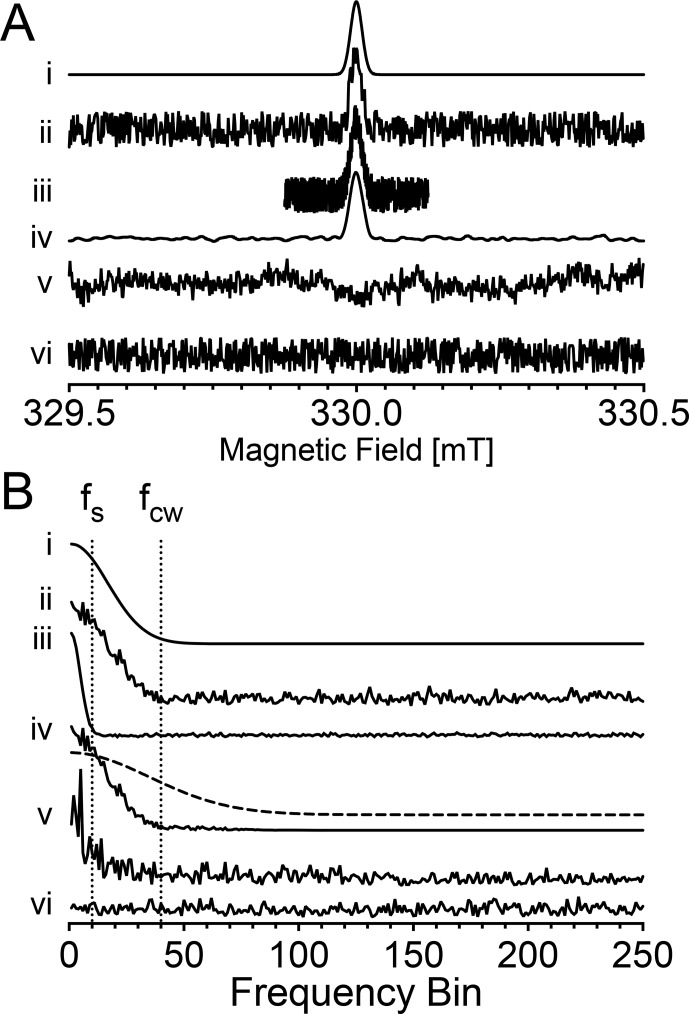
Simulated field and calculated corresponding Fourier transform employed to illustrate the details of spectral bandwidth. Simulated data are plotted in **(A)** field space and **(B)** truncated Fourier space to provide sufficient detail. Plotted is a (i) noiseless Gaussian spectrum, (ii) Gaussian spectrum with additive white noise, (iii) oversampled Gaussian spectrum with additive white noise ( OSR=4 ), (iv) Fourier-filtered Gaussian spectrum with Gaussian filter (dashed), (v) pink noise with noise floor, and (vi) white noise.

SNR is improved in oversampling by reducing the spectral bandwidth by the oversampling ratio (OSR) and effectively spreading noise power spectral density by the same amount; see Fig. A1 in the Appendix (ii and iii) (Oppenheim et al., 1999). Since segmented magnetic resonance spectra are collected, a further decrease in the spectral bandwidth is exhibited since only a fragment of the spectrum is sampled. Ultimately, the bandwidth of the noise increases, but it is the reduction in the bandwidth of the signal (due to the collection of partial spectra) that is reduced, allowing a more aggressive filter without spectral broadening.

A key advantage of the segmented acquisition strategy is that it allows the whole spectrum to be oversampled far beyond what the spectrometer hardware permits in a single continuous sweep. Legacy instruments such as some Bruker E500 consoles are limited to 8192 points per continuous sweep. For example, with SOFFA, collecting a spectrum with a 2 mT segment sweep width, a 0.1 mT field step size, 4096 points per segment, and 100 steps produces 204 800 effective data points across the whole spectrum. Because the magnetic resonance signal is stationary and time-invariant, the signal contribution from every segment that covers a given field position is fully correlated, meaning the same EPR line appears at the same field value in each overlapping segment. The noise, however, is independent during each acquisition and is therefore uncorrelated between segments. The result is a whole-spectrum dataset with correlated signal and uncorrelated noise, which is precisely the condition that enables effective Fourier filtering and signal averaging to improve the SNR ratio.

Another advantage of oversampled segmented data collection has to do with noise that is loosely correlated to the signal or noise that is colored, such as 1/f noise. In conventional CW, the time constant acts as a low-pass filter on the data, while the sweep duration acts as a high-pass filter on the noise. Repeating full-spectrum sweeps for signal averaging introduces 1/f noise that cannot be directly filtered without broadening the EPR signal. Furthermore, because this noise is time-correlated, traditional signal averaging becomes less effective, improving the SNR ratio by less than the ideal $\sqrt{m_{a}}$ factor. However, by reducing the sweep into segments and maintaining the sweep rate (e.g., 60 s for 10 mT and 12 s for 2 mT ; 0.167 $\mathrm{mT}\;s^{-1}$ ), the shorter observation window inherently rejects ultra-low-frequency drift. Furthermore, due to the segment step timing and excessive overlap, any remaining correlated noise is randomized between segments. This decorrelation allows the signal averaging to approach the theoretical white-noise limit of $\sqrt{m}$ .

Once each of the segments is sufficiently oversampled, filtering can be employed to reduce the high-frequency content outside the spectral bandwidth by applying finite-impulse response (FIR) block-filtering techniques.

### 3.2 Fourier block-filtering each segment separately

The SOFFA data acquisition method differs from typical Fourier block-filtering based on two important factors: (i) each segment is a new collection of data with field- or frequency-stepped correlated signal and uncorrelated noise, and (ii) each segment overlaps the next segments for a total of m overlapping segments; see Fig. 1. Due to oversampling, the spectral bandwidth of the spectrum in each segment is reduced; see Fig. A1, comparing spectra ii and iii.

**Figure 3:**
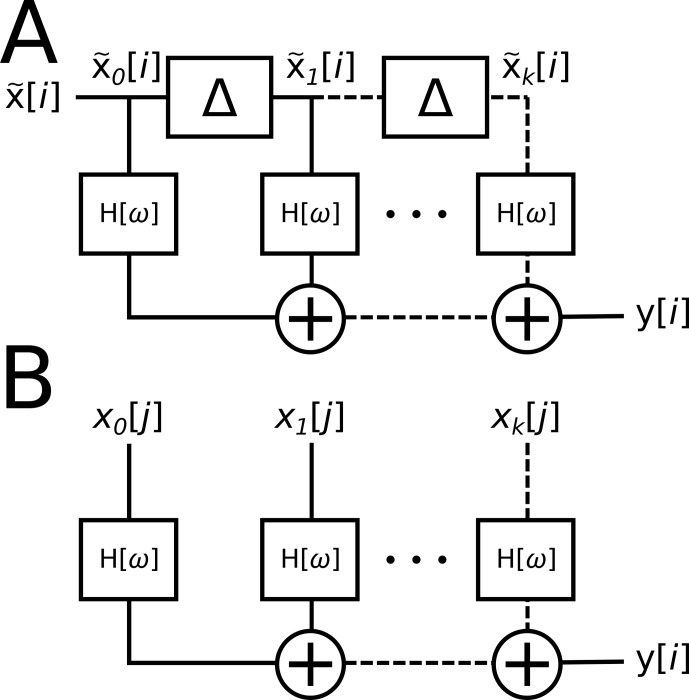
**(A)** The overlap-add filter design is used for a large time domain signal x(t) , where it is discretized $\tilde{x}[i]$ and segmented by delayed blocks Δ . Each discretized window is Fourier filtered by H[ω] and concatenated. **(B)** In a segmented magnetic resonance experiment, each overlapped segment has noise that is uncorrelated. Each segment is discretized separately and Fourier filtered by H[ω] , and overlapping segments are further averaged during concatenation. Without overlap ( L=1n ), the methods of **(A)** and **(B)** are equivalent.

Fourier block-filtering is illustrated in Fig. 3. Each segment is convolved with the filter H[ω] , such that

5 $${\text{G}}_{k}[\omega ]={\text{Y}}_{k}[\omega ]{\text{H}}_{k}[\omega ],$$

where ${\text{Y}}_{k}[\omega ]$ is the Fourier transform (Fourier space ω ) of the discrete signal, and the convolution is ${\text{G}}_{k}[\omega ]$ . In this work, a fixed FIR Gaussian filter is used, defined by

6 $${\text{H}}_{k}[\omega ]=\mathrm{exp}\left(\frac{-(\omega -\gamma {)}^{2}}{2\sigma_{n}^{2}}\right),$$

where ω is the discretized frequency, and γ in the central point in the Fourier-transformed data and has a variance of $\sigma_{n}^{2}$ . The smallest variance $\sigma_{n}^{2}$ , which shows no spectral broadening, is chosen for each L . However, it should be noted that these filters are fixed in this work but, in principle, can vary at each k value or be more sophisticated, such as in adaptive averaging techniques (Cochrane and Lenahan, 2008; Manning et al., 2020).

Spectral leakage occurs when the chosen Gaussian standard deviation $\sigma_{n}$ is more narrow than the spectral bandwidth of the desired signal. As the length of L is increased for each segment, the OSR must be increased to maintain the same spectral bandwidth. However, in practice, this is impractical, and, instead, the standard deviation of the filter must be modified to ensure no spectral leakage. This is visually evidenced by a broadening of the linewidth and the reduction in features of the EPR spectrum. By choosing to adjust $\sigma_{n}$ , the spectral bandwidth will approach that of the whole spectrum as L approaches the total length of $\tilde{x}[i]$ ( i=0,1,…,N ), and the SNR improvement will approach that of filtering after concatenation, as employed by Hyde et al. (Hyde et al., 2013).

Since the optimum windowing method for the SOFFA algorithm is rectangular, abrupt discontinuities may exist in field space as the data are collected segmentally. Discontinuities cause Sinc-like features in Fourier space. However, Fourier filtering each segment with an FIR Gaussian filter removes these discontinuities and produces smooth overlap and concatenation. Filtering each segment separately removes the need for filtering the periodic noise associated with the step, as in Hyde et al. (2013), which is required if the spectrum is filtered after concatenation.

As m increases, the length of the tail further overlaps with the head of multiple segments, resulting in averaging.

### 3.3 Segment-overlap averaging

If a signal input is infinitely long or for signals that need to be filtered in real time, the overlap-add method is used with short-time Fourier transform (STFT) block-filtering for efficient digital signal processing (Oppenheim et al., 1999; Ifeachor and Jervis, 2002). Block-filtering breaks a long or continuous signal x(t) into a series of spectral windows with a fixed discrete length. Each window is filtered individually in Fourier space and concatenated back together, as illustrated in Fig. 3A. Finally, the output of block-filtering and recombining the data with an overlap-add or overlap-save method is equivalent to filtering the long-signal x(t) data directly. In the case of the SOFFA method, illustrated in Fig. 3B, each component is acquired separately.

For overlap-add processing in SOFFA, a high-resolution overlap-add grid is constructed at 4 times the target resolution, with 4P evenly spaced points spanning the full field range t . Each acquired segment $x_{k}[j]$ is mapped onto this grid by computing the field position $f_{k,j}=F_{\text{first}}+k\cdot s+\delta_{j}$ for each point j , where $\delta_{j}$ is the field offset of point j within the segment sweep. The value $x_{k}[j]$ is accumulated into the nearest grid bin, and a count is incremented. After all K segments have been processed, each bin is divided by its count to yield the averaged value, and bins with no contributions are filled by linear interpolation from neighboring bins. This accumulation and averaging step is where the segment-overlap averaging takes place on the fine grid.

Once each segment is successfully filtered, the segments must be concatenated together. Due to the use of a rectangular window, the segments can be concatenated in Fourier space to save processing time, and a single inverse Fourier transform (IFFT) is needed to obtain the discretized spectrum. Because the data are zero-filled and aligned, this is done simply by summation

7 $${\text{y}}_{\text{tot}}[\omega ]=\text{IFFT}\left(\sum_{k=0}^{K-1}{\text{Y}}_{k}[\omega ]{\text{H}}_{k}[\omega ]\right).$$

Since each of the segments has a portion of the signal and non-coherent noise that lies within the spectral bandwidth (low frequency), an SNR improvement of $\sqrt{m}$ is expected by averaging the overlapped segments.

When a fixed filter (i.e., the same filter used on each segment) is employed and each block is recombined using the overlap-add or overlap-save algorithm, the SNR of the filtered signal is exactly equivalent as if the significantly oversampled whole signal was processed directly.

Finally, the first and last L points are called the head and tail, respectively. Since the head and tail do not have a complete segment-overlap dataset, they must be removed. As m increases, the head and tail regions may encroach on the magnetic resonance signal. In practice, for large values of m , the total sweep width should be increased to accommodate for the removal of the head and tail region.

### 3.4 Decimation

The total number of points in the final concatenated spectrum $y_{\text{tot}}[i]$ is a function of the oversampling rate, the step size, and the number of steps. To generate a SOFFA spectrum with a conventional number of output points P (for example, 512, 1024, 2048, or 4096), the filtered grid is decimated to P output points using a moving-average window, where each output point is the mean of a fixed number of neighboring fine-grid points centered on the corresponding output field position. This produces the final output spectrum y[p] , p=0,…,P-1 . The decimation step conserves the SNR gains achieved during Fourier block-filtering and segment-overlap averaging. In addition, the moving-average window acts as a final low-pass filter on the fine grid while reducing the number of points to a standard (typically 1024).

### 3.5 Implementation

The block-filtering approach employed by the SOFFA method provides flexibility in filter design. If the filter H[ω] is constant, as in this work, the output y[i] is equivalent whether block-filtering is used or the oversampled data are filtered after concatenation but before decimation. One must be careful to always filter before decimation to not reduce the filtering effectiveness. If the filter varies per segment ${\text{H}}_{k}[\omega ]$ then filtering after concatenation is no longer equivalent (Lin and Mitra, 1996; Karami and Saarnisaari, 2018). There is no direct analogue in conventional CW averaging to segment-specific filtering because a full sweep is processed as a single globally averaged trace, whereas SOFFA permits each independently acquired segment to be denoised with its own locally appropriate filter, for example by applying a Wiener filter separately to each segment before recombination (Oppenheim et al., 1999).

In general, the SOFFA method splits data processing into two complementary operations: (i) high-frequency filtering and noise shaping and (ii) low-frequency averaging.

#### 3.5.1 Segmented continuous-wave EPR

A SOFFA-CW experiment is performed by collecting oversampled data from a standard lock-in phase-sensitive detector over a fraction of the spectrum and systematically stepping the center field to obtain the full signal. In practice all parameters kept the same as the traditional CW experiment. The number of points is set to 4096 or 8192, the time constant is significantly reduced to minimize lock-in noise correlation, and scan rate should be adjusted to optimize the oversampled segments to ensure no broadening from passage. One rule of thumb is to use the same sweep rate as the traditional experiment and to make the time per Gauss constant over the truncated segment (e.g., 60 s for 10 mT and 12 s for 2 mT ; 0.167 $\mathrm{mT}\;s^{-1}$ ). The spectral bandwidth of a CW experiment is larger compared to the partial line shapes acquired with SOFFA, allowing for improved filtering of the high-frequency noise.

#### 3.5.2 Segmented non-adiabatic rapid scan

In SOFFA-NARS the continuous field sweep of CW is replaced by a stationary field position at which a large-amplitude, slow-rate sinusoidal or trapezoidal modulation drives the spin system through resonance repeatedly. Because $dB_{m}/dt$ is slow enough that the spin system remains in thermal equilibrium throughout, no passage effects occur, and all harmonics of the modulation frequency are collected coherently by a digitizer, yielding a pure-absorption spectrum after processing (Kittell et al., 2011; Hyde et al., 2013; Kittell and Hyde, 2015). The static field is then stepped, and the process is repeated across the full spectrum.

This architecture cleanly replaces the CW sweep rate constraint with two independent parameters: modulation amplitude, which sets the spectral window sampled at each field position and therefore maps directly onto the segment width g , and modulation frequency, which determines both the time resolution per cycle and the number of modulation cycles collected per segment. Longer dwell time per field position means more cycles, more comb filtering, and a lower noise floor at the cost of the total experiment time.

The key advantage over SOFFA-CW is that the step size s and modulation amplitude (segment size g ) can be reduced independently of any magnet sweep rate or hysteresis constraint since the magnet is stationary during acquisition. This removes the hardware floor on sweep time as the binding limit and shifts the practical ceiling to the modulation frequency stability and digitizer dynamic range instead.

The step size should be set such that significant overlap occurs ( L>20n ). For NARS spectroscopy, pure absorption is directly collected, providing the optimal spectral bandwidth for SOFFA. Multiple time averaging (MTA) is standard in NARS due to the rapid data collection rate and filters low-frequency and shot noise associated with environmental sources (Wilmshurst, 1990; Eaton et al., 2014). No change in the NARS collection procedure is required to implement SOFFA-NARS processing.

#### 3.5.3 Frequency-stepped NMR

Frequency stepping is used to obtain broadband (Sanders et al., 2018) or ultra-wideband (Schurko, 2013) NMR spectra for exotic (Ash and Grandinetti, 2006; Massiot et al., 1995; Lipton et al., 2002) and quadrupolar nuclei (O'Dell et al., 2009), including applications with magic-angle spinning (Pell et al., 2013). A narrow-band variant of this approach, in which the frequency is stepped to produce a series of segmented spectra, is compatible with the SOFFA method.

#### 3.5.4 General usage and parameter selection

Collection of SOFFA data requires the ability to step one parameter in known increments and relate these to a fixed number of points per segment and also requires that the signal of interest is stationary and repeatable in that parameter. Table 2 compares the parameters for a conventional EPR CW experiment and a SOFFA-CW experiment. A traditional CW spectrum should first be collected to determine the full magnetic field sweep width of the EPR signal. In modern EPR software (e.g., Bruker XEPR, Bruker MS5000, SpecMan), it is straightforward to derive the required parameters from the configured number of points.

**Figure 4:**
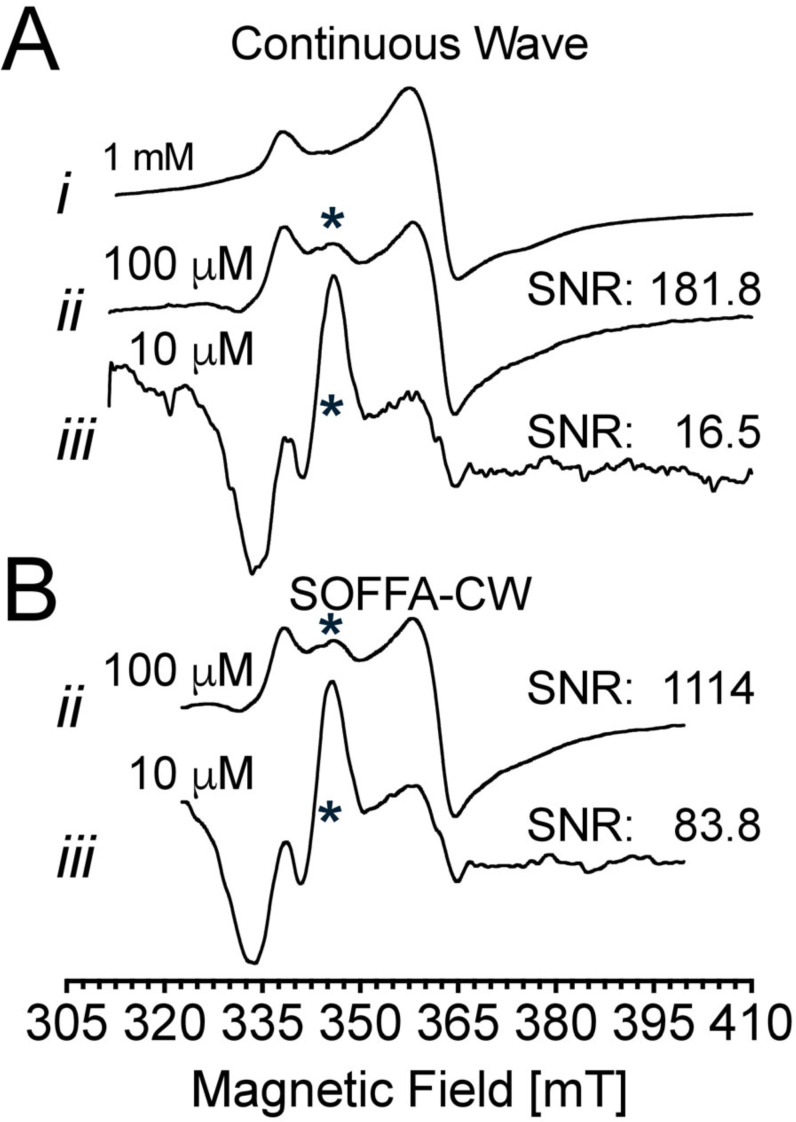
CW spectra were collected on a Bruker E5180 X-band bridge with a dielectric resonator (9.70 GHz) of the reduced $[4\mathrm{Fe}\text{-}4S{]}^{+}$ of apo [FeFe]-hydrogenase from *Chlamydomonas reinhardtii* at 1 mM, 100 µM , and 10 µM concentrations – spectrum i, ii, and iii, respectively. The reference spectrum of 1 mM concentration was collected over 100 mT , 4 min scans averaged nine times with 4096 points. The traditional CW experiment with 100 µM concentration was collected over 100 mT , 4 min scans averaged 25 times with 4096 points for a total time of 100 min. Both CW spectra were filtered with a $\sigma_{n}=75$ . The SOFFA-CW experiment with 100 µM concentration was collected in 0.5 mT steps of 25 mT over 30 s and 4096 points for a total time of 100 min. The SOFFA-CW experiment with 10 µM concentration was collected in 0.5 mT steps of 25 mT over 30 s and 4096 points for a total time of 100 min. For both SOFFA-CW experiments, a total of 200 steps were collected over a 100 mT total sweep. All segments were block filtered with a σ=50 and with an m=48 . All spectra were collected at a temperature of 15 K and at an incident power of 0.63 mW , with a field modulation amplitude of 0.5 mT . The EPR feature at 340 mT ( ∗ ) is a background caused by an unknown radical in the shield of the MD5.

For a SOFFA-CW experiment, a separate experimental configuration is created, starting at the lowest magnetic field. In the example of Fig. 4, the SOFFA-CW segment sweep width is set to one-quarter of the total sweep (25 mT ), with 4096 points per segment. The time constant should be set to the minimum available value to avoid pre-filtering within each segment. The experimental scan time per segment (30 s) is set to 1/200 th of the total CW experiment time to match the total SOFFA-CW acquisition time. Further averaging of the full SOFFA-CW dataset is always possible; however, the overlap between segments already provides built-in averaging (overlap m=48 in this example).

In the ProDEL code^1^, three variables need to be set: the starting field (one step below the lowest field) is placed in value, the final field is placed in endParValue, and the segment step is placed in parStep. Bruker XEPR then executes the ProDEL script on the active experiment. The data are saved by XEPR, and the acquired segments are loaded into the Python GUI^1^, and the filter variance is adjusted to ensure no spectral broadening.

**Table 2:** Comparison of parameters required for a traditional CW experiment and a SOFFA-CW experiment. The traditional time constant (RC filter) should be set as low as possible for SOFFA-CW. Total scan times do not include overhead (e.g., field flyback time, program latency).

	Traditional CW	SOFFA-CW
Sweep width ( t )	100 mT	100 mT
Power	0.6325 mW	0.6325 mW
Mod. amp.	0.5 mT	0.5 mT
No. of pts ( N )	4096	4096
Scan time ( τ )	240 s	30 s
Aves.	25	1
Time constant	58.59 ms	–
Filter ( σ )	–	50
Seg. width ( g )	–	25 mT
Seg. step ( s )	–	0.5 mT
Total exp. time	t× aves.	τ× (t s^−1^)
	6000 s	6000 s

The SOFFA method is equally applicable to spectra with well-resolved, narrow hyperfine lines. Three parameters govern performance in this regime. First, the Gaussian filter variance $\sigma_{n}^{2}$ must be minimized relative to the intrinsic linewidth to prevent spectral broadening. Second, the field step size s can be reduced to increase the overlap factor m and thus the number of averaged segments; on the Bruker E500, s is practically limited to 0.01 mT and conservatively to 0.025 mT by the field controller precision and magnet hysteresis. Fine adjustment of the effective averaging is therefore most conveniently achieved by varying s , while larger gains are better obtained by repeating the full segment stack (repetitions). Third, the per-segment scan rate should not exceed the effective rate of a conventional full-sweep experiment: for example, a 10 mT sweep collected over 60 s implies that each 2 mT segment should require at least 12 s.

For a fixed Gaussian filter, the combined SNR improvement of the SOFFA method is the product of the oversampling gain and the segment-overlap averaging gain $\sqrt{m\cdot \text{OSR}}$ . Substituting m=g/s and OSR=(t/g)⋅(L/N) , the product is simplified to

8 $$\sqrt{m\cdot \text{OSR}}=\sqrt{\frac{g}{s}\cdot \frac{t}{g}\cdot \frac{L}{N}}=\sqrt{K\cdot \frac{L}{N}},$$

and so the theoretical upper bound on SNR improvement is $\sqrt{K\cdot L/N}$ , the square root of the total number of acquired data points across all segments normalized to the conventional spectrum length. This is equivalent to the SNR that would be achieved by collecting a single continuous sweep of K⋅L points in the same total experiment time and represents the limit at which SOFFA converges to conventional filtered averaging. This assumes that the filtering, measurement time, and decimation are equivalent.

Three practical constraints prevent this limit from being reached in full. First, the minimum magnet step size s (conservatively 0.25 G and at best 0.1 G on the Bruker E500) places a ceiling on K as set by the field controller precision and magnet hysteresis. Second, the per-segment sweep rate must remain below the threshold for adiabatic passage effects, which constrains how rapidly the field can be stepped through any given segment. Third, 1/f noise imposes a lower bound on useful segment length: if segments are shortened beyond the correlation length of the low-frequency noise, the decorrelation assumption underlying the $\sqrt{m}$ averaging gain begins to break down. Within these hardware constraints, SOFFA approaches but cannot exceed the SNR of a hypothetical instrument with no step size or memory limitations. For a fixed filter, the hardware is the ceiling, not the algorithm.

An additional advantage of segmented data collection is noted. By breaking the acquisition into rapid, localized segments, the SOFFA algorithm acts as a hardware-level high-pass filter that systematically prevents the long-term accumulation of 1/f -correlated low-frequency drift.

For strictly white noise, once the oversampling ratio and filter are aggressive enough to reject all out-of-band noise down to negligible levels (while still preserving the physical EPR lineshape), any further increase in the OSR or decimation factor provides no additional benefit. One cannot filter out noise that resides within the exact same frequency band as the EPR signal without destroying the signal itself; that is left to averaging, which scales by $\sqrt{m}$ .

By restructuring the acquisition chain, the algorithm successfully decouples high-frequency filtering from low-frequency averaging. The high-frequency broadband noise is aggressively filtered out at the local segment level by leveraging the massive oversampling density, while the low-frequency 1/f drift is mitigated through the rapid scanning and subsequent spatial averaging of the overlapping segments. Because these two distinct noise domains are handled by two separate, optimized mechanisms, the method provides a data collection scheme that drastically suppresses correlated noise and extends sensitivity toward the limits imposed by practical hardware step size, lock-in amplifier memory limits, and sweep-rate constraints.

## 4 Results and discussion

The following experiments were tested using the SOFFA method described above.

### 4.1 Continuous-wave EPR

A series of CW EPR spectra of apo [FeFe]-hydrogenase from *Chlamydomonas reinhardtii* of varying concentrations were collected using a bismuth germanate ( ${\mathrm{Bi}}_{4}({\mathrm{GeO}}_{4}{)}_{3}$ , BGO) dielectric resonator retrofitted in a Bruker MD5 housing (Ivanov et al., 2016). The apo protein has one reduced $[4\mathrm{Fe}\text{-}4S{]}^{+}$ cluster with an effective S=1/2 . Three concentrations of 1 mM, 100 µM , and 10 µM were prepared and confirmed with UV-VIS by measuring the characteristic 400 nm feature indicating relative amounts of [4Fe-4S] (Knörzer et al., 2012).

The 100 µM CW spectrum yielded a SNR of 181.8, while the SNR of the 10 µM CW spectrum was calculated to be 16.5. For SOFFA-CW, an SNR improvement of 6.1 ( 1114:181.8 ) is exhibited between the 100 µM CW experiment and the SOFFA-CW experiment. On the other hand, the SOFFA-CW experiment with 10 µM concentration had an SNR ratio that was calculated to be 83.8 and an SNR improvement of 5.1 ( 83.8:16.5 ). An average of 5.6 increase in concentration sensitivity is shown for the two concentrations using SOFFA-CW.

### 4.2 Non-adiabatic rapid scan

**Figure 5:**
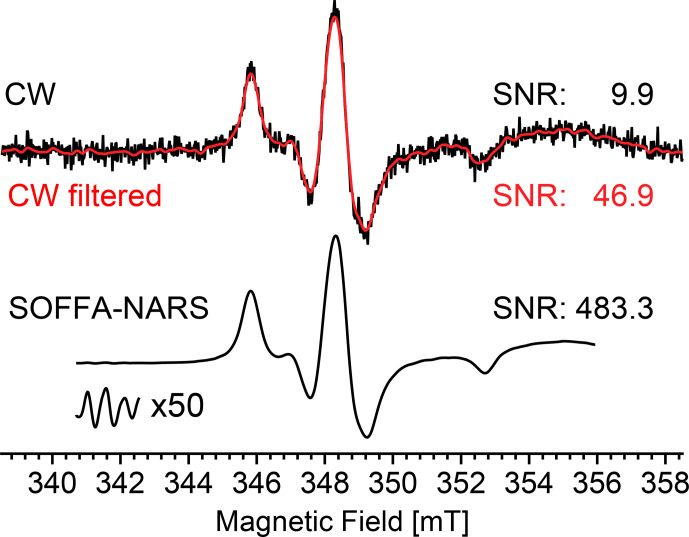
A 150 µM site-directed spin-labeled hemoglobin sample in 82 % glycerol at 18 °C is collected with conventional-CW EPR in a five-loop–four-gap loop–gap resonator at X-band (9.81 GHz) using 0.1 mT field modulation at a rate of 100 kHz and a 5 ms time constant, with 16 averages over 20 mT (2 min scan) at 1 mW of microwave power, yielding an SNR of 9.9. A Gaussian filter is applied to the CW data, yielding an SNR of 46.9. SOFFA-NARS data are collected with 0.025 mT steps ( s ), an overlap of m=40 using a trapezoidal field ramp of 11.33 kHz with a 50 µs flat region, and an amplitude of 0.5 mT . Data were acquired over 95 % of the trapezoidal sweep in one direction. Processing the data with the SOFFA method results in an SNR of the pure-absorption signal (not shown) of 3890. An MDIFF pseudo-modulation of 0.1 mT field modulation equivalent was used to display the spectrum in a more conventional form, yielding an SNR of 483.3. The zoomed inset is the first 500 points of the NARS signal. Data were collected on a custom-built X-band bridge dedicated to segmented-NARS spectroscopy. An NARS dispersion signal was also collected (not shown). Both experiments took approximately 32 min each.

Conventional CW data are compared to SOFFA-NARS of a 150 µM site-directed spin-labeled hemoglobin sample in 82 % glycerol (18 °C ) at X-band (9.81 GHz), shown in Fig. 5. Experimental parameters are stated in the figure caption. This sample is in the slow-tumbling regime of approximately $15\times 10^{-9}\;s$ rotational correlation time $\tau_{\text{corr}}$ . After 16 averages of 2 min scans, a CW SNR of 9.9 is realized. Adding a Gaussian filter increases the SNR by a factor of 4.7 ( 46.9:9.9 ). By processing the segmented-NARS data with the SOFFA filtering method, the collected pure-absorption signal has an SNR of 3890 (data omitted). In order to display the spectrum in a more conventional form, the first derivative is computed using an MDIFF pseudo-modulation of 0.1 mT . An SNR increase of 10.3 ( 483.3:46.9 compared to the filtered CW data) was achieved for the same measurement time of 32 min. The zoomed inset is the first 500 points of the NARS signal multiplied by a factor of 50. Since NARS uses a balanced mixer and an A/D converter, the dispersion signal was also collected for no increase in measurement time (data omitted). The NARS linear background was baseline-corrected in Xepr.

### 4.3 Practical considerations

The SNR ratio of EPR is fundamental to the usefulness of the technique. Improvements in EPR fall into three categories: technical developments, such as resonator and spectrometer advances; multiharmonic data collection (Yu et al., 2015); and advanced averaging and filtering techniques, such as, wavelet analysis (Srivastava et al., 2016) and adaptive averaging (Cochrane and Lenahan, 2008; Manning et al., 2020) techniques. One must take care that any new method does not over-filter and deliver a distorted EPR spectrum.

In addition, the evaluation of new technology must be done with both white noise and correlated 1/f noise under consideration. White noise has a flat power spectral density across all frequencies with statistically independent samples, plotted in Fig. A1B spectrum vi. Correlated noise (“pink” or 1/f noise), shown in Fig. A1B, spectrum v, concentrates power at low frequencies that directly overlap with the EPR signal bandwidth, making it impossible to filter without distorting the spectrum. The 1/f noise power spectral density is a function of scan time and is therefore a measure of instrument stability (Kittell et al., 2011), and changing the sweep speed shifts the correlated noise frequency content relative to the EPR signal bandwidth.

**Figure 6:**
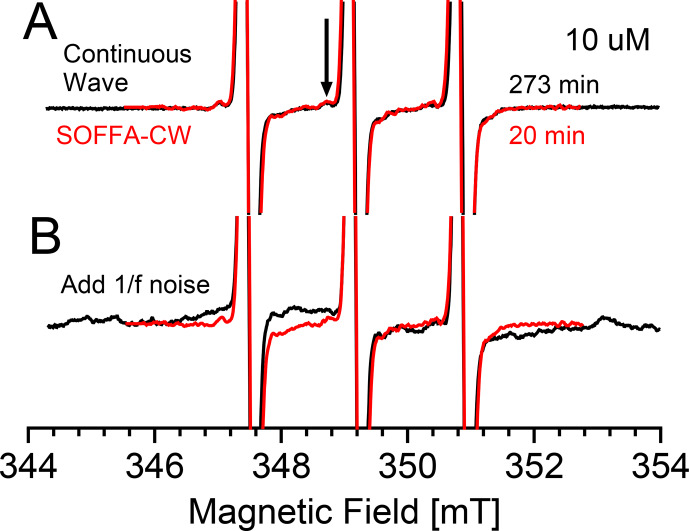
A 10 µM TEMPO spectrum was recorded using **(A)** traditional CW averaging for 273 min (400 averages; black) and SOFFA-CW for 20 min (red). One ${}^{13}C$ feature is highlighted. The continuous-wave experiment was collected with a 10 mT sweep of 1024 points over 41.94 s with 400 averages, a 20.48 ms time constant at 3.2 mW , and 0.1 mT field modulation at 100 kHz for an SNR of 1146. SOFFA-CW was performed with a 3 mT sweep of 8192 points over 10.39 s, with 100 steps of 0.1 mT each, a 1.28 ms time constant at 3.2 mW , and 0.1 mT field modulation at 100 kHz for an SNR of 1218. **(B)** Averaged 1/f for 400 averages, added to the continuous-wave data (black; SNR of 205); 1/f was added onto each of the 100 steps of the SOFFA-CW (red; SNR of 768).

For this experiment, the data are plotted in Fig. 6A; the ${}^{13}C$ lines of 10 µM TEMPO have been highlighted by using traditional averaging, shown in black, by scanning 400 times over a 273 min time period for an SNR of 1146. In contrast, the same SNR ratio can be achieved in only 20 min (red) without loss of the subtle ${}^{13}C$ lines by using the SOFFA-CW method for an SNR of 1218.

Since the power spectral density of 1/f noise is similar to the EPR signal, both correlated noise and EPR signal have a similar shape in the field domain, making fitting subtle spectral features difficult without significantly improving the SNR ratio of the EPR signal with hardware improvements since excessively long averaging will not average out 1/f noise.

To isolate performance under strictly controlled 1/f drift conditions, a hybrid empirical-simulated approach was utilized. Specifically, the empirically acquired EPR spectra shown in Fig. 6A were corrupted by mathematically injecting randomly generated 1/f noise into the signal. The noise was implemented in the frequency domain following a 1/f power law. The resulting spectrum for the continuous wave is shown in black in Fig. 6B.

For SOFFA, the randomly generated correlated noise was added to each of the 100 segments, and averaging was performed through the SOFFA algorithm. Since the continuous-wave EPR experiment and the SOFFA-CW experiment have different sweep times (41.49 and 10.39 s, respectively) the frequency content of the correlated noise is different, and this is taken into account in this approach. The resulting spectrum for SOFFA-CW is shown in red in Fig. 6B. From this, it is clear that the subtle ${}^{13}C$ lines of the 10 µM TEMPO are lost in the continuous-wave EPR spectrum and remain in the SOFFA-CW due to the reduction in SNR from 1146 to 205 when 1/f noise was added. The SOFFA method demonstrates enhanced EPR spectral features by not only enhancing the SNR ratio by a factor of 3.7 compared to traditional continuous-wave measurements ( 767:205 ) but also effectively mitigating 1/f correlated noise, thereby preserving subtle spectral features such as ${}^{13}C$ lines and enabling more reliable spectral fitting even in challenging low-concentration samples where 1/f starts to dominate due to averaging requirements.

Finally, the version of Bruker Xepr (v.2.6b-160) used to collect the SOFFA spectra is not optimized for segmented data acquisition. Future work will implement segmented-NARS using the Xepr Python API interface to decrease the latency associated with large datasets. An SNR improvement is expected by increasing the number of averages for the same total data collection time. Despite current software limitations, the spectra collections shown in Figs. 4 and 6 were performed on a commercial Bruker E500 instrument with no hardware modifications. Further improvement in segmented-NARS is expected by using trapezoidal sweeps and processing both the sweep-up and sweep-down data (Kittell and Hyde, 2015; Tseytlin, 2017).

## 5 Conclusions

The SOFFA data acquisition method, introduced herein for segmented magnetic resonance spectroscopy, has been shown to decrease low-frequency background signals and significantly improve the SNR ratio by effectively splitting high-frequency filtering and low-frequency averaging.

The improvements described here are phenomenological: the SOFFA method is built upon known DSP foundations, and the SNR gains are demonstrated experimentally rather than derived from a unified analytical model. A quantitative first-principles treatment (one that predicts the net SNR improvement as an explicit function of overlap factor m , oversampling ratio (OSR), filter variance $\sigma_{n}^{2}$ , and noise color) is the subject of ongoing work. Nevertheless, the experimental data presented across three distinct EPR measurement conditions consistently demonstrate the practical value of the approach across biologically relevant samples and measurement conditions. The ProDEL script and Python GUI are publicly available to facilitate adoption.^1^

The use of the block-filtering approach employed by the SOFFA method provides a flexible framework for implementing complex filter designs. For example, frequency content could be analyzed, and an appropriate filter could be chosen block-wise ( $H_{k}(\omega )$ where k=0,1,…,K ; illustrated in Fig. 3) to maximize SNR while minimizing spectral broadening. Future work studying the effects of adaptive filtering and “spectral sensing” is underway in order to be able to choose filter parameters without a priori information (Karami and Saarnisaari, 2018; Nguyen-Thanh and Koo, 2013). Additionally, the SOFFA method lends itself to more advanced techniques, such as wavelet analysis and filtering (Srivastava et al., 2016) or sub-band-multirate filtering (Vetterli, 1987), which may yield improved SNR and can be customized with a priori or real-time iterative calculation of the frequency content of the spectrum. Combining advanced filtering techniques with adaptive averaging (Cochrane and Lenahan, 2008; Brinton and Hirsh, 2010; Manning et al., 2020) of the overlapping segments provides a powerful state-of-the-art digital signal processing toolkit for segmented spectroscopy. The SOFFA algorithm also lends itself to multi-harmonic detection without modification (Hyde et al., 1990; Tseitlin et al., 2011).

It should be noted that the first field-stepped rapid-scan (RS) spectra were obtained by Eaton and colleagues (Yu et al., 2015). However, segmented-RS differs from the enabling field-stepped RS spectra described therein. The work of Yu et al. (2015) stepped the field while the whole RS spectrum was excited and then averaged together. Potentially, for rapid scan, deconvolution of each segment block-wise before filtering is feasible (Tseytlin, 2017). Future work will explore these possibilities.

As frequency-swept NARS and RS are more widely adopted, the SOFFA filtering technique introduced here may provide an additional benefit by filtering and averaging signals from the frequency response of microwave components which create large and often quadratic backgrounds (Hyde et al., 2010; Tseitlin et al., 2011; Strangeway et al., 2017). By using the off-resonance frequency or field sweep in the NARS data collection, the large static frequency response can be background-subtracted from the whole dataset before processing without adding significant overhead.

Implementing SOFFA-CW on a standard Bruker E500 spectrometer and collecting segmented CW is straightforward and demonstrates an increase in concentration sensitivity of 5.6 for the same measurement time. In a SOFFA-NARS experiment, a factor-of-10.3 SNR improvement, shown in Fig. 5, is achieved with no change in the experimental procedure described in the literature (Kittell et al., 2011; Hyde et al., 2013; Kittell and Hyde, 2015).

Finally, it should be noted that the theoretical limit of Eq. (8) derived above assumes a fixed Gaussian filter applied uniformly across all segments, and, therefore, assuming no hardware limitations, SOFFA is mathematically equivalent to filtering a single continuous sweep of equivalent length. This effective oversampling makes the SOFFA algorithm especially important for hardware with a fixed maximum number of points. However, this ceiling is specific to that fixed-filter case and does not represent a fundamental bound on the method. Once the filter $H_{k}[\omega ]$ is allowed to vary per segment, the analogy to a single continuous sweep breaks entirely: there is no continuous-sweep equivalent to a matched or adaptive filter tuned to the local noise statistics of each independently acquired segment. This is not merely a practical distinction because the independent acquisition of each segment is precisely the condition that makes per-segment adaptive denoising statistically valid. Furthermore, the same shortened observation window that decorrelates 1/f noise between segments also provides the locally stationary noise estimate that adaptive filtering requires, meaning the two benefits share a common origin.

The collection of segments therefore simultaneously decouples correlated low-frequency noise and unlocks a class of sophisticated denoising strategies with no corollary in conventional continuous-sweep spectroscopy. As adaptive and matched filtering techniques are incorporated into the SOFFA framework, the upper bound on SNR improvement will no longer be set by hardware step size constraints but by the sophistication of the per-segment denoising strategy itself.

## Data Availability

The ProDEL script and Python GUI used to collect and process SOFFA data are available at https://github.com/jsidabras/SOFFABruker (last access: 30 June 2026; 10.5281/zenodo.20766183, Sidabras, 2026).

## Appendix A Shape of noise

Plots of simulated Gaussian spectra in field and Fourier space are shown in Figs. A1A and B, respectively. A noiseless Gaussian is shown in Fig. A1, spectrum i, and white noise is added to make the SNR approximately 4, as shown in Fig. A1, spectrum ii. By filtering the noisy spectrum with a Gaussian filter (Fig. A1B, dashed line) the noise outside of the spectral bandwidth ( $f_{\text{cw}}$ ) can be removed, as shown in Fig. A1, spectrum iv. However, by oversampling, shown in Fig. A1 spectrum iii, the spectral bandwidth is reduced ( $f_{s}$ ) by the oversampling factor OSF, and the noise power spectral density is reduced by the same amount. In order to mimic the methodology of oversampled segmented-data collection, the number of points remains the same, but the collected data are only a segment with a quarter of the field sweep.

An example of pink noise is shown in Fig. A1, spectrum v. The power spectral density, shown in Fig. A1B, illustrates the challenge with filtering pink noise: the concentration of power spectral density in the same region of the spectral bandwidth. On the other hand, white noise, shown in Fig. A1 spectrum vi, has a flat power spectral density response.
